# A scoping review of health models for the community mental health needs of the United Arab Emirates: Nurturing the social determinants of mental health through social prescribing in the Middle East

**DOI:** 10.12688/f1000research.153262.3

**Published:** 2024-10-08

**Authors:** Richard Mottershead, Sadeq AL-Fayyadh, Nabeel Al-Yateem, Muhammad Arsyad Subu, Wegdan Bani-Issa, Mohamed Hassan Taha, Fatma Refaat Ahmed, Jacqueline Maria Dias, Shukri Adams, Adil Farooq Wali, Ghada Shahrour, Abbas Al Mutair, Conrad Murendo, Nafi Alonaizi, Ali Alhaiti, Majed Mowanes Alruwaili, Abeer Nuwayfi Alruwaili, Jim McManus

**Affiliations:** 1 Department of Nursing, College of Health Sciences, University of Sharjah, Sharjah, United Arab Emirates; 2 College of Nursing, University of Baghdad, Baghdad, Iraq; 3 College of Medicine and Medical Education Centre, University of Sharjah, Sharjah, United Arab Emirates; 4 College of Nursing, Ras Al Khaimah Medical and Health Sciences University, Ras Al Khaimah, United Arab Emirates; 5 College of Pharmacy, Ras Al Khaimah Medical and Health Sciences University, Ras Al Khaimah, United Arab Emirates; 6 Faculty of Nursing, Jordan University of Science and Technology, Irbid, Irbid Governorate, Jordan; 7 Research Center, Almoosa Specialist Hospital, Al-ahsa, Saudi Arabia; 8 Save the Children International, Kabul, Afghanistan; 9 Medical Services, Military Medical Services, Riyadh, Saudi Arabia; 10 Department of Nursing, College of Applied Sciences, Almaarefa University, Riyadh, Saudi Arabia; 11 Nursing Administration and Education Department, College of Nursing, Jouf University, Sakaka, Saudi Arabia; 12 National Director of Health and Well-being, Public Health Wales, London, UK

**Keywords:** mental illness, community mental health, health models, scoping review, social prescribing, social determinants of mental health, United Arab Emirates, Middle East.

## Abstract

Despite the growing interest in social prescribing the diversification of health and social care strategies to support the well-being of patients has remained entrenched with a focus on the hospital setting within the Middle East. The United Arab Emirates has commenced progressing community mental health care to lead changes in how care and treatment are delivered within the United Arab Emirates.

The authors adopted the use of the framework of Arksey and O’Malley (2005) to provide a systematic approach to searching the literature and creating a comprehensive foundation to guide the review. This scoping review provides a better understanding of the compatibility, content and outcomes of a selection of health models whilst also allowing further clarification before empirical studies. The scoping review findings will inform the proposed use of social prescribing as an actionable approach to create a focus on the need to include and empower the social determinants of mental health. This article proposes an evidence-based health strategy that supports and enhances recent additions to national legislation on the inclusion of the Mental Health Law within the United Arab Emirates to meditate and prevent inequities in addressing the mental health needs of citizens and residents within the nation’s diverse communities.

## Introduction

Individuals with mental health needs encounter challenges in their abilities to handle everyday tasks throughout the course of their illness. The authors explain that mental health care delivery within the United Arab Emirates (UAE), continues to be predominantly delivered via a traditional in-patient model with opportunities for the development of community mental health services. This view is shared by The World Health Organisation ( WHO, 2017) explains that this overreliance of hospital-based care has the potential to create barriers for allowing families to be involved in the care and treatment which subsequently may affect recovery from mental illness. Dattani (2021) provides great insight in explaining that reported mental health conditions in the Middle East have remained relatively consistent over the past two decades. However, mental health conditions are increasing as a share of the total disease burden. Al Habeeb et al. (2023) states that collectively, the Middle East and North Africa (MENA) region forms the global concentration for the proportion of mental health disorders as a share of the total disease burden. Dattani (2021) in agreement, highlights that in Qatar, Kuwait, Oman, and Jordan the share of mental health conditions as a share of the total disease burden is over double the global average of 5%. The study’s authors urge caution in that the post-COVID-19 era has created an imperative for healthcare providers to continue to explore new innovative strategies to address the mental health needs of a population having experienced a global humanitarian crisis. This correlates with Al Habeeb et al. (2023) that the young population may be at further risk of mental illness and a higher burden of noncommunicable diseases. Far from a want of being the harbingers of doom, the authors highlight hope in that there is indeed, an increased focus regionally on mental health by policymakers and there are clear signs of progress across the Middle East.

Globally, there has been a progression away from long-term mental health hospitals, due in part to isolation and limited and ineffective treatments that did not address the full range of SDMH. The WHO ( Singh, 2021) identifies that there was a progression for best practice towards integrated health systems oriented towards health and social care arrangements in the community setting. The noted sociologist Erving Goffman (1963) noted the historical negative impact of institutionalization within the seminal work on mental health institutions, referred to as Asylums. Since that time period there has been an agenda to replace full reliance on the mental institutions and instead include community centres, outpatient facilities and mental health provision in general hospitals. This initiative is on-going and preserves is with international agenda’s such as the WHO comprehensive mental health action plan 2013-2030: We must rise to the challenge ( Singh, 2021). Within the MENA region there has still been a lingering reliance on in-patient care, and this has been self-reported in Saudi Arabia and Jordan where there are noted opportunities to progress community care. The WHO (2020) reports that in Jordan 49% of patients in mental hospitals remain for five years or more and a further 17% for between one and five years. Al-Diyab ( WHO, 2020) Saudi Arabia’s director general of mental health reported his concern that, of the then 4,000 beds available in mental hospitals, 1,000 were blocked by patients who could not leave as their families would not accept them back. Whilst, hard to digest within a region that is admired for its strong family support and compassion, it is to be understood when paralleled to Goffman’s work (1961) and regional literature on the presence of stigma towards those suffering with mental illness ( Zolezzi et al., 2018). Goffman widened the understanding of deprivation approaches by describing the progression away from institutionalisation as a series of ‘degradations’ and a process of ‘mortification’, in which an individual’s identity and personal characteristics are eroded over time due in part to a lack of access to their community and family (1961). Recognizing this need for an expansive approach to community care the Emirates Health Services (EHS) has launched an initiative to develop a network of primary mental health clinics in the community, strengthening community mental health services across the UAE ( WAM, 2020).

The author’s purpose is to review health models from different countries as found within the literature with correlation to the best practices towards community care. This paper highlights social prescribing as an alternative treatment strategy to the traditional biomedical models embedded within the over reliance of the mental health hospitals. Social prescribing is advised to assist individuals to connect and thrive within their communities and to support improved health and well-being as linked to the social determinants of mental health. The authors highlight possible challenges towards the dissemination and implementation of the identified health models before concluding the paper.

## Objective and review question

The author’s rationale for conducting a scoping review was to provide a methodology for determining the state of the evidence on the selected topic, this is especially useful when issues require clarification before empirical studies are undertaken. Therefore, the purpose of this study is to adopt Arskey and O’Malley’s scoping review methodology to determine the following with respect to identifying appropriate health models to support SDMH and the adoption of social prescribing:

1. What is the extent and nature of published scientific literature on health models including the research designs used, areas of clinical practice, and compatibility for use of social prescribing to address SDMH?
2. To what extent do the identified models address the core components of community care needs in the UAE and support the adoption of the proposed community focused treatment strategy of social prescribing to provide a holistic review of SDMH?

A secondary purpose of this study was to reflect on the depth and breadth of evidence surrounding challenges of establishing new care strategies for community care by charting the published systematic reviews on historical hurdles to paradigm shifts in care provision. This review was conducted as a secondary procession to identify evidence for policy makers to consider when seeking to enhance client-centered frameworks by adopting new health models since effective care is the most definable and consistent component of care in the community.

## Methods: Scoping review

This study undertakes a scoping review of the relevant literature on models of health and selects those that have an evidence base to address clients and societal needs of SDMH. The models, if adopted could enable the use of social prescribing within community settings to support the healthcare systems within the UAE. Where possible the authors will seek to highlight modifications to identified health models to illustrate how these models can be modified to meet the study’s aim.

The use of scoping reviews is advocated by Arksey and O’Malley (2005). They explain that the use of a scoping review can highlight if there is a need to address broader topics where many different study designs might be applicable and whether there is a challenge in identifying a clear question. Arksey and O’Malley (2005) further explain that whereas literature and systematic reviews are predominantly concerned with providing answers to questions from a relatively narrow range of quality assessed studies, scoping reviews are less inclined to seek to address a specific research question nor, consequently, to assess the quality of included studies. This was of particular interest for this study as the establishment of the UAE’s first community mental health services as the authors interest to create a treatment strategy that address SDMH inclusive of medical strategies needed a review strategy that was broad, evidence based and yet, capable to identify capable and effective health models.

The study adhered to the suggested framework of Arksey and O’Malley (2005). This framework provided a systematic approach to searching the literature and also aided in establishing a comprehensive foundation to guide the review. There was a need by the authors to determine the extent of research previously conducted on the identified models and to highlight any grey literature, theory or perspectives that may have relevance to the understanding of the selected topic a sit applied to the culture and the people of the Emirates. Table 1 provides a tabular format for the study’s adherence to this framework. The authors needed to adapt the model proposed by Arksey and O’Malley but this has some similarities to the experiences of Colquhoun et al. (2014). These authors emphasised that a scoping review is not a linear process but rather describes a cyclical process of the researcher (authors) going back-and-forth between early findings and new emerging insights. Therefore, the authors made changes in the search terms and even the questions. Hence, the sections not being sequential with the identified six stages of the Arksey and O’Malley (2005) framework.

**Table 1.** Scoping Review Framework - Six Step Approach.

1. Identify the Research Questions 2. Identify the Relevant Studies 3. Study Selection 4. Charting the Data 5. Collating, Summarising and Reporting 6. Consult Stakeholders and Policy Makers – Aim: To obtain more references, provide insights on what the literature fails to highlight (Optional).

Arksey and O’Malley (2005).

This research strategy of adopting a scoping review, rather than a systematic review allowed the authors an evidenced based approach when sourcing relevant studies that identified multiple health models. Arksey and O’Malley (2005) advocate that an inclusive approach should be adopted. This allowed the authors to review qualitative, quantitative and randomised control trials within the scoping review. However, it became apparent that there was a challenge in sourcing health models that had been adopted within the Middle East, inclusive of adhering to SDMH principles.

The co-authors were selected as they are embedded within diverse clinical practices within the UAE, Iraq, Jordan, Indonesia, Egypt, Pakistan, Saudi Arabia, Afghanistan, Nigeria, and the UK. These co-authors are themselves published within research pertinent to meeting the objectives e.g., Abdalla et al. (2024). As highlighted by Grant and Booth (2009) scoping reviews can negate systematic reviews typical critical appraisal for quality and risk of non-inclusion. The geographical dominance of the medical model as opposed to SDMH meant that the co-authors were crucially aware of gaps in knowledge on this specific topic. The authors wished to better understand the types of research carried and to triangulate relevance to the cultural nuances of the MENA region. Therefore, the authors needed to extrapolate and establish a new paradigm and justify the need to offer a different perspective and healthcare strategy. This scoping review reviewed the evidence from published research and identified the profile and key themes relating to SDMD and possible use of social prescribing. Models were analysed and identified in conjunction with the cultural nuances of mental healthcare in the region. This allowed for comparisons to be made from randomised controls trials and analysis found in both quantitative and qualitative global studies.

### Applied search strategy

The scoping review within this study incorporated the 2 identified questions to explore the aim of this study: What evidence is there of appropriate health models that can be adopted to use social prescribing to treat mental illness and address SDMH within the community care setting. The search determined that there had been regional interest in social prescribing within the United Kingdom (UK), Europe and the United States (US) and that this had created a wide-ranging source of research and literature on social prescribing ( Morse et al., 2022). What was less evident was a review of health models to implement this strategy in community mental health care settings within the Middle East. This necessitated a need for the authors to undertake a broader and wider focus with a flexible hierarchy of evidence to identity relevant literature. The scoping review extrapolated the evidence from published literature and identified the profile and key themes aligned to the needs assigned to a proposed health model. The health models were analysed and identified in conjunction with the relevance of and success of their implementation on community settings, alongside information gained from an evidence base for their application to the ethos of social prescribing as an empowering and alternative as a non-pharmaceutical focused treatment scheme that could be implemented within UAE society and culture.

Key databases searched were: CINAHL, MEDLINE, Cochrane Library, ASSIA, Web of Science and Scopus. The database search was also supplemented by hand searches of relevant publications/policy documents located within the University of Sharjah libraries. All considered literature was considered key to underpinning and providing an evidence base for this review article. No time restriction was set initially, as the authors wanted to seek relevant literature that may be seminal and illuminated the historical significance of the evidence base presented. However, for specific studies relating to health models and social prescribing, a secondary literature search was undertaken from the time periods 1940’s to 2024 to capture the most current research. A wide range of studies were sought that focused on policy objectives and initiatives influenced by international and national health agenda, through to independent and statutory studies exploring relevant health models, social prescribing and SDMH. A wide variety of search terms were used independently or in different combinations which were directly pertinent to the research questions or were slightly tangential to the research questions, this was to ensure that any emergent sources that may be linked to seminal literature might be found, and their relevance explored to identify innovative new healthcare strategies and or approaches.

### Ethics

The study has conducted a scoping review of existing relevant studies and will not recruit any participants directly; thus, ethical approval was not required.

## Results

### Self-Management and Self-efficacy within the Inclusion of Health Models for Community Care

In following six-step approach the authors identified 22 possible models. There was limited published information within the MENA region but extrapolation from international studies allowed the authors to consult stakeholders and policy makers (step-six of the adopted scoping review strategy of Arksey and O’Malley, 2005). This consultation identified the importance of self-management given the comparable limitations of mental health provision within the region and early stage of development of community mental health services. Within the review of literature and consultation, the authors identified the concept behind self-management as mentioned previously to be self-efficacy ( Liu et al., 2023). It is accepted that individuals experiencing mental ill-health may have different symptoms that directly affect their cognitive ability to engage in society. Therefore, managing the symptoms of mental health is extremely important. In coping with such conditions, healthcare providers within the community must empower clients and let them be positively involved in addressing their SDMH through an individualized plan of care. Williams et al. (2007) highlights that self-management education programs are significant for empowering clients to acquire new skills in order to enhance the self-care of their conditions. In reviewing the literature of the evidence base of health models the authors ensured that self-management and self-efficacy were adopted conceptually as gatekeepers for the inclusion of any identified models. Four noteworthy types of models identified by the authors are the Health Belief Model, Motivational Interviewing Health Promotion Model and finally the Crescent of Care Nursing Model. These four models can be utilised with minimal involvement of the traditional bio-medical model practices arguably entrenched within the region’s healthcare practices. These suggested models of care align with a social prescribing framework and could be applied to health policy directives within the region.

### Identified Health Models for the Application of Social Prescribing to meet the Social Determinants of Mental Health Care needs of the UAE

**Health belief model**

The Health Belief Model (HBL) was developed by Hockbaum, Leventhal Kegeles and Rosenstock in the 1960’s. The model was identified within the scoping review as it has relevance as it creates a focus for the client to be able to make choices on their own health care needs ( Rosenstock, 1966). This active decision-making approach was based on the seminal psychological theories first identified and explored by Lewin (1944), who proposed that individuals assign value to the potential outcome, this therefore influences the selection of health behaviours based on their belief of the results of the desired outcome.

Later studies by Wallston (1992) and Schenkman et al. (2008) demonstrated the benefits of this framework for health promotion for those with heath needs. Their research has relevance for this study in that their findings indicate that the HBM, as a socio-cognitive approach that could support the mental health needs of individuals. In applying this model to social prescribing, the authors propose that individuals might be likely engage in the selected psychosocial interventions prescribed if they have the mindfulness that the given health-related treatment has a positive impact on their activities of daily living, when they believe the intervention will be effective, and when they perceive few limitations to participating and taking empowering action. The authors believe that the HBM could provide a structure to developing and evaluating social prescribing in the UAE when applied to community mental health schemes as there is an enhanced emphasis on improving mental health awareness as applied to the causalities of the social determinants of mental health and associated well-being of people in the UAE.

### Motivational interviewing

First devised by Millar (1983) and then expanded by Millar and Rollnick (1995) motivational interviewing (MI) has been recognized as highly effective in the treatment of mental illness. MI was first devised for the treatment of addictions and alcohol misuse as a directive counselling approach. From its conception there have been encouraging results demonstrating its effectiveness in promoting change in health behaviour and client led lifestyle changes. A key concept of MI is that it seeks to delve into the individual’s ambivalence not to promote healthy change and seeks to alter these maladaptive responses ( Levensky et al., 2007). When exploring this model for the use of social prescribing within community mental health settings, there is strong evidence for this client-centered approach but does rely on the client’s intrinsic motivation to change and so there would be a holistic effort from all multi-disciplinary teams to have a familiarity and confidence in applying MI within the community setting. This model follows four key counseling principles ( Rollnick, Miller, Butler, 2023; Levensky et al., 2007) which the authors have reviewed and adapted to the focus of this study:

The adapted model of Rollnick, Miller, Butler (2023) and Levensky et al. (2007) by this study’s authors provides a proposed framework for the use of social prescribing in the community mental health setting that focuses on and seeks to impact the social determinants of mental health. The framework relies on the practitioner’s interpersonal style ability to engage in reflective listening, asking open-ended questions, affirming, and summarizing their engagement with their client. This approach could instill motivation to change in the client by creating alternative cognitive realities of their life post engagement with MI ( Table 2).

**Table 2.** Motivational interviewing to support SDMH.

Processes	Objectives	Questions to address	Social Determinants of Mental Health
**Engaging**	Strengthen the link, show empathy and interest	What is the actual reality of the individual?	Create an awareness of the individual’s relationship to income, wealth, education, employment, social support, environment, ethnicity, gender, sexuality, social systems.
**Focusing**	Create a focus to ensure that the discussion targets change within the life of the client.	What should we address as a target of change?	Create an empathetic focus on the key SDMH key points (above) and motivation to implement change.
**Evoking**	Objective 1: Reasons and Abilities to Change (the importance of change) Objective 2: Change talk (the confidence to change)	How relevant would it be to go towards change? What abilities and strengths does the individual have to get to the target of change?	Creating an empowering framework for graduating change as it applies to the identified SDMH needs.
**Planning**	Engagement talk. How to change.	How will the individual get there.	Formulating identified psychosocial interventions through social prescribing to assist improved well-being and health.
**Evaluation of Practice**	Re-evaluation of effectiveness	What went well, what can be improved?	Explore enhancements to SDMH and evaluate impact of social prescribing plan.

### Health Promotion Model

Historical conceptions on health promotion were predominantly focused on supporting the individual with healthcare needs to help themselves make lifestyle changes ( Minkler, 1989). However, as discussed by Whitehead (2004) there has since been an evolving dominance of socio-political emphasis in health promotion which has overtaken a former emphasis on individualistic and behaviour focused care. The authors highlight the ability of the health promotion model to assist with the transformative process of developing community mental health services in the UAE as the model has an evidence base within the socio-political of health promotion activities. Therefore, the model has an evidence base to develop and reform social structures through developing participation with social prescribing with representative stakeholders from multiple agencies and disciplines. However, caution should be taken as the model has historically incurred criticism that with the focus on individual responsibility for health can contribute to victim blaming ( Hancock, 1986). Early studies by Robertson and Minkler (1994) acknowledged that health promotion recognized the social, economic and political determinants of health with a later inclusion of social justice as noted by Carlisle (2000).

The authors determine that the health promotion model could create a workable framework by which the ecologically driven socio-political-economic determinants of health are addressed as they impact on community mental health. However, the authors acknowledge that this model does not have a specific focus on religion and spirituality. As emphasized by Lovering (2008) there is a need to create an emphasis on the importance on Muslim faith as it applies to health in countries in the Middle East. Therefore, the authors expanded the parameters of the scoping review and identified the following model for inclusion in this study.

### Cresent of Care Nursing Model

**Figure 1.**
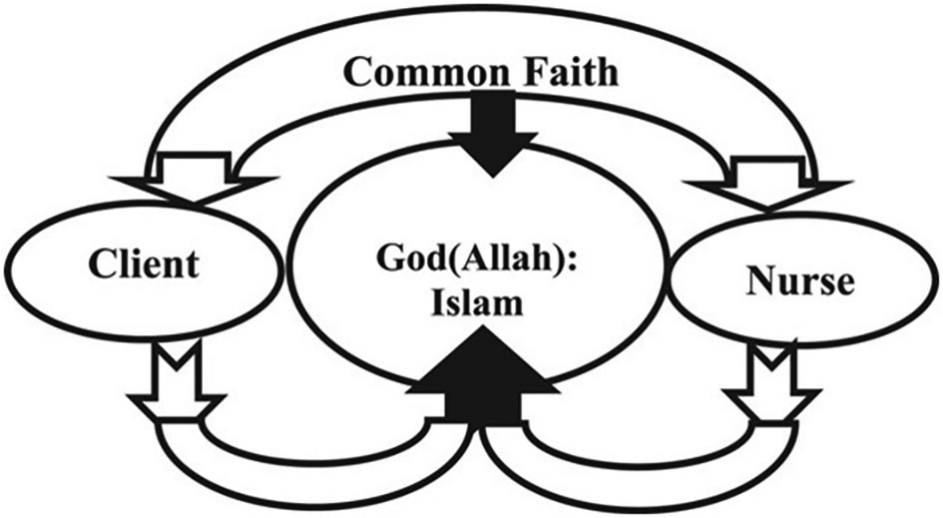
Spiritual Care Model Crescent Care Model.

The Crescent of Care nursing model is derived from an ethnographic study of the health beliefs and care meanings of Arab Muslim nurses caring for Arab Muslim patients in the Middle East region by Lovering (2008). The model is a culturally sensitive and holistic approach to the spiritual, cultural, psychosocial, interpersonal, and clinical caring needs of Arab Muslims within the UAE and the wider MENA region. Lovering (2008) demonstrates the linkage of professional nursing values and combines them to creation of this model. The model creates a central organizing point with the client and their family as the focal point. Shared meanings on spirituality between the nurse, patient and family nurture meaningfulness and enhance care. The authors remonstrate that this model links into the SDMH as it is set within a context of psychosocial, cultural, interpersonal, clinical, and spiritual elements of care by respectfully acknowledging that an Arab conceptualization of health is derived from the religion of Islam. The wisdom of including this model within this scoping review is the presence and relevance of the concept of tawhid. Kounsar (2016) describes tawhid as a oneness with Allah and requires that a Muslim lives in a way that demonstrates a unity of the body and mind with Allah. Lovering (2008) writes that Tawhid implies therefore that there is no separation of the body from the spiritual dimension of health. Kounsar (2016) highlights central beliefs to Islam that Muslims also believe. This ethos can be applied to seeking to address the SDMH via social prescribing as illustrated within the following model ( Figure 1) used by Al-Fayyadh et al. (2022) to illustrate Lovering’s Cresent of Care Nursing Model:

This model is based on Al-Fayyadh et al. (2022) interpretations of Lovering’s Crescent Care Model which supports clients to understand and appreciate the link between short and long-term goals. This paper suggests that this approach is driven by religious and cultural sensitivity that may enhance motivation for the client and so support them adhering to the psychosocial interventions identified as part of social prescribing. The authors suggest that the inclusion of a focus on the ethos of tawhid may promote continued involvement in goal setting, active participation, and enhanced responsivity in that the clients take responsibility for their own health and empowered care through social prescribing. The interplay via shared belief can foster and promote effective and culturally and religiously nuances psychosocial intervention that the client wholeheartedly embraces through tawhid. As in all changes, there are inherent challenges to progress which will be discussed now.

## Discussion

### Future Considerations: Stigma and Habitus

Identified challenges exist in developing workforces capable of mental health within communities setting and independent from the current institutional arrangements that do not meet all SDMH. Caution must be taken not to replicate errors and delays away from institutionalized care which can isolate clients away from families and their communities, making reiteration all the more difficult. The authors believe that there is a lack of mental health awareness due to engrained practices (habitus) are further compromised with the presence of stigma within health systems not utilizing health models that seek to enhance the quantity and quality of care. Such issues have existed in much of the world and should not be seen as solely regional concerns. The papers finding indicate that opportunities exist for shifts in treatment provision as suggested by the inclusion of social prescribing to aid services to meet the full range of SDMH. The inclusion of mental health strategies and laws herald improvements with a recognition of the likely benefits of integrating mental healthcare delivery with community care and the management of common mental health conditions indicating encouraging changes to current practices.

The World Health Organisation (2022) estimates that the majority of individuals with mental illness do not seek treatment, citing reasons as concerns a perceived damaging of their family’s reputation, standing within the community, opportunities for marriage, encountering discrimination, exclusion from society, and stigma. Consequently, these individuals experience diminished self-esteem ( Stuart et al., 2019) and poor academic achievement ( Bruffaerts et al., 2018).

Goffman (1963, p. 3) describes stigma as “an attribute that is deeply discrediting. It diminishes the social status of an individual by reducing them in the eyes of others from being whole and usual to tainted and discounted”. Stigmatization has the potential to diminish an individual’s self-esteem, cause friction within familial units, and have an impact on their prospects by negatively affecting their employability ( Picco et al., 2019). Within a previous study by Mottershead and Ghisoni (2021) it was proposed that social prescribing is an empowering strategy to assist individuals to connect and thrive within their communities and supports improvements in their health. This has also been observed within the Middle East within a study by Mottershead and Alonaizi (2022) and Mottershead (2022) in exploring social prescribing use with addiction and substance misuse treatment strategies. Whilst further research is required, it may be theorized that there is an associated stigma towards mental illness that still creates challenges for treatment within a public and community setting. Again, noted by Mottershead and Alonaizi (2022) was that whilst, social prescribing schemes have demonstrated a reassuring evidence base and continue to gain popularity, there has been little evidence of its use within the Middle East. A question, must there be contemplated – why?

This article argues that stigma alone is not the causality but rather that healthcare systems have these become transfixed on hospital-based care for mental illness due decision making predominantly focusing on the foundation of medical services since the conception of the UAE in 1971. This is in no way a criticism, few countries have achieved the success of the UAE in healthcare, but a realization might acknowledge a concept that Weber, Husserl and later Bourdieu (1990) identified as ‘Habitus’. It has been seen in other areas of research as argued by Professor Loader at the University of Oxford ( Loader, 2007) that society can directly impact the formation of services. It is feasible that a symbiotic relationship may be experienced to a general public’s sensitivities (stigma) towards mental illness and the ability of a health services to create new strategies and treatments. Habitus, entails competence and know-how, it demonstrates great skill and awareness of how to achieve success through a pre-determined set of strategies, policies and procedures almost effortlessly through reflexivity ( Bourdieu, 1990).

As the reference to ‘dexterity’ suggests, entails competence and know-how. It captures the skilled activity of the expert player rather than the conditioned response of the lab rat. The footballer who moves instinctively into the right position on the pitch, arriving just as the ball does and at exactly the right angle to it to put it in the back of the net, all without having to think reflectively about doing so, exemplifies this, as Bourdieu’s (1990). However, as was seen during the Covid pandemic, preexisting systems either failed or had to be modified, innovation became the lifeblood of a healthcare ability to become resilient and to adapt. To this end, the article has explored the available published scientific literature on health models including the research designs used and assed their credibility for clinical practice to support social prescribing and to address SDMH. In addition, the diverse international spectrum of co-authors and engagement with stakeholders has allowed further dialogue on the adoption of the proposed community focused treatment strategy of social prescribing to provide a suggested examination of SDMH within the UAE and wider Middle East healthcare practices. The authors argue that further research should follow to allow a wider contemplation of the SDMH, and how social prescribing can support the evolvement of current practices that utilize treatments pre-dominantly focused on pharmaceutical interventions for mental illness.

## Conclusion

With 21 ^st^ century life impacting on well-being there is an imperative to adopt health models with an ethos of self-management as enshrined within social prescribing. The article has presented for consideration, four self-management models with a theoretical evidence-base that could be applied to care for those experiencing mental illness within the community setting in the UAE and wider Middle East region. The focus on the SDMH via social prescribing links into the agenda of the UAE Centennial 2071 (2018). The author’s present these four models emphasizing their benefits; however, a challenge is identified in the need to ensure that health insurance recognizes the importance of social prescribing and empowers clients to select this as a health delivery option. Indeed, the models within their empowering components of self-efficacy demonstrate potential as they mirror the ethos embedded within social prescribing, yet as identified by the authors this does not necessitate success for performance attainment. Motivational Interviewing is still relatively unknown outside of a rapidly developing mental health service. The Health Promotion Model, lacks an emphasis of religious awareness and this is limiting within communities so attuned to faith and their spirituality. The Crescent of Care Nursing model has a strong evidence base from Saudi Arabia. UAE is an inclusive and tolerate multi-faith community that may require inclusive insights guided by Islam and embracing of other practices. Likewise, the Health Belief Model may require bespoke alterations to attune to the regional healthcare landscape. Although, challenges are present, the study demonstrates that if there is to be a provoking of new healthcare strategies, then a focus on SDMH will create a sustainable community mental healthcare strategy, empowering clients, and acknowledging the significant importance of the inclusion of families within the recovery and care of mental illness.

## Data Availability

No data are associated with this article.
