# Supplementary material for: A scoping review of health models for the community mental health needs of the United Arab Emirates: Nurturing the social determinants of mental health through social prescribing in the Middle East
Source: F1000Res. 2024 Oct 8;13:841. Originally published 2024 Jul 26. [Version 3] doi: 10.12688/f1000research.153262.3 (PMC11626703; doi:10.12688/f1000research.153262.3)
Supplement: Peer review history

# Reviewer report: Reviewer response for version 3

**DOI:** 10.5256/f1000research.172688.r329823

**Published:** 2024-12-04

**Authors:** Gary Lamph

**Affiliations:** 1 Keele University, Keele, England, UK

I am now happy to approve this manuscript based upon the changes made and the detailed author responses to my original review.

Are the rationale for, and objectives of, the Systematic Review clearly stated?

Yes

Is the statistical analysis and its interpretation appropriate?

Yes

If this is a Living Systematic Review, is the ‘living’ method appropriate and is the search schedule clearly defined and justified? (‘Living Systematic Review’ or a variation of this term should be included in the title.)

Yes

Are sufficient details of the methods and analysis provided to allow replication by others?

Partly

Are the conclusions drawn adequately supported by the results presented in the review?

Partly

Reviewer Expertise:

Mental Health Nursing Research

I confirm that I have read this submission and believe that I have an appropriate level of expertise to confirm that it is of an acceptable scientific standard.

# Reviewer report: Reviewer response for version 2

**DOI:** 10.5256/f1000research.170940.r320404

**Published:** 2024-09-26

**Authors:** Gary Lamph

**Affiliations:** 1 Keele University, Keele, England, UK

This paper outlines a scoping review which examined health models for community mental health needs of the UAE.  It well written but I agree with the 1st review is lacking in some places detail we would expect to see.

 The rationale for the use of scoping study methodology could be improved. Ordinarily a scoping study is typically used when there is a dearth of literature in the field, or to complete some broad mapping of the literature. Could the authors add this justification as otherwise it feels like the methodological approach has just been chosen randomly, and I don’t think current justification is strong enough in the opening abstract but is clearer in the main text.  I also would expect to see how the research team applied each stage of the scoping study review this could be achieved by adding a table of the stages and how they attended to each stage.

 Introduction feedback

 Please review and rephrase as this sentence could be viewed as contradicting itself in suggesting prevalence is stable then its increasing?  ‘…provides great insight in explaining that although the prevalence rates of mental health conditions in the Middle East have remained relatively consistent over the past two decades, mental health conditions are increasing as a share of the total disease burden’

 2nd Paragraph of Introduction – I think authors could add here a sourced reference that mentions how also some of the movement away from inpatients is also aligned to more cost effectiveness. Authors do make reference to other important factors such as institutionalisation but if this could be added, I think it could be complimentary to this background.

 In the papers objective and review question section – your choice of scoping study methodology is better explained with reference to ‘clarification before empirical studies’, I don’t think in the opening abstract this is captured though.

 Page 5 – I find it unusual not to see search terms or indeed a Prisma diagram can these be provided? How many included paper etc?  This would be complimentary to the paper.

 Results Section.  I feel here authors jump into reporting results, could an opening statement or paragraph of the key areas of results be provided to open up this section and then the provision of the details across the 4 models presented.

 I think that a discussion section relating to the results and findings collectively is required.

 Overall, this is a well written paper in large parts, but I think lacks some of the methodological detail I would expect to see. Stages of Scoping Review and how it is applied and the absence of  search terms and paper inclusion / exclusion criteria are missing and hit results. It is also missing the process used for determining what was included and rejects.  A Prisma diagram would somewhat attend to this and a write up of the process would be beneficial, this could be added in table form.

 I think the results section requires some reorganisation to flow properly for the reader with a reformat and new subheadings.

 I would suggest that Self-Management and Self-efficacy within the Inclusion of Health Models for Community Care – becomes an opener for the results section. This would then lead onto the  four models you will be reporting on from the literature. Each of the 4 models should then be clearly sub-headed and follow on as they do, but I would suggest you share how many papers informed each sections and hence are reported on in each section.

 Finally Future Considerations: Stigma and Habitus, I would suggest could become your discussion section and where you report upon what you found that aligns to the 2 objectives. I think objective 1 is met via the paper, but I am not sure you have from the findings reported on how this meets objective 2 fully.  Discussion needs strengthening to answer the paper objectives fully.

 Conclusion Section - Some of the conclusion details could go into the discussion and I would suggest a briefer conclusion to highlight headline findings.

 I hope my reflections are useful for consideration if the above are attended to satisfactory. I think this is worthy of publication if the above can be satisfactorily addressed as its content is of interest and adds something new to the literature.

Are the rationale for, and objectives of, the Systematic Review clearly stated?

Yes

Is the statistical analysis and its interpretation appropriate?

Yes

If this is a Living Systematic Review, is the ‘living’ method appropriate and is the search schedule clearly defined and justified? (‘Living Systematic Review’ or a variation of this term should be included in the title.)

Yes

Are sufficient details of the methods and analysis provided to allow replication by others?

Partly

Are the conclusions drawn adequately supported by the results presented in the review?

Partly

Reviewer Expertise:

Mental Health Nursing Research

I confirm that I have read this submission and believe that I have an appropriate level of expertise to confirm that it is of an acceptable scientific standard, however I have significant reservations, as outlined above.

# Response

**Published:** 2024-09-28

**Authors:** Dr. Richard Mottershead

**Affiliations:** College of Health Sciences, University of Sharjah, Sharjah, Sharjah, United Arab Emirates

*FYI -*

 Reviewer Comments are highlighted in **"Bold"**

 Author responses are denoted by "R1, R2, R3" and so on"

**This paper outlines a scoping review which examined health models for community mental health needs of the UAE.  It well written but I agree with the 1st review is lacking in some places detail we would expect to see.**

**The rationale for the use of scoping study methodology could be improved. Ordinarily a scoping study is typically used when there is a dearth of literature in the field, or to complete some broad mapping of the literature. Could the authors add this justification as otherwise it feels like the methodological approach has just been chosen randomly, and I don’t think current justification is strong enough in the opening abstract but is clearer in the main text.  I also would expect to see how the research team applied each stage of the scoping study review this could be achieved by adding a table of the stages and how they attended to each stage.**

 R1.Thank you for your comments. The authors added a section on page 6 providing an additional rationale and justification for adopting the use of a scoping review and the use of Arskey and O’Malley’s (2005) 6 step approach following feedback from reviewer 1. We have added additional commentary on this within page 6. We believe that this along with the present Table on p.6 supports your request. As you rightly highlight a scoping review is used when there is a dearth of data. Our article focuses on the Middle East. We used that the exploration of SDMH and social prescribing are advancing within the UK, yet there are still issues of inertia and hence, relatable and culturally nuanced literature to drawn upon. As we identify within the methods section a scoping review allowed us to have a broader in our search and exploration of this subject within the MENA region.

**Introduction feedback**

**Please review and rephrase as this sentence could be viewed as contradicting itself in suggesting prevalence is stable then its increasing?  ‘…provides great insight in explaining that although the prevalence rates of mental health conditions in the Middle East have remained relatively consistent over the past two decades, mental health conditions are increasing as a share of the total disease burden’**

 R2.Thank you for your insight and the sentence has been rewritten to clarify the point of the original author Dattani (2021).

**2nd Paragraph of Introduction – I think authors could add here a sourced reference that mentions how also some of the movement away from inpatients is also aligned to more cost effectiveness. Authors do make reference to other important factors such as institutionalisation but if this could be added, I think it could be complimentary to this background.**

 R3. This is an interesting observation, and the authors thank the reviewer for allowing them an opportunity to highlight a significant difference between service delivery strategies/capabilities. The countries (UAE) ability to build infrastructures, buildings is significant. Building costs are lower than that of e.g., UK. A decision to expand community services within the country is not based on cost saving (indeed it may well be more cost effective to build another inpatient hospital) but rather enhanced patient care and recovery across the nation’s 7 Emirates.  

**In the papers objective and review question section – your choice of scoping study methodology is better explained with reference to ‘clarification before empirical studies’, I don’t think in the opening abstract this is captured though.**

 R4. Thank you for this suggestion on how we may guide the reader to understand our rationale for selecting a scoping review. We have included your suggestion within the abstract.  

**Page 5 – I find it unusual not to see search terms or indeed a Prisma diagram can these be provided? How many included paper etc?  This would be complimentary to the paper.**

**Results Section.  I feel here authors jump into reporting results, could an opening statement or paragraph of the key areas of results be provided to open up this section and then the provision of the details across the 4 models presented.**

 R5. Thank you for your observation. As you may be aware Arksey and O’Malley (2005) original framework for conducting scoping reviews has since been further developed and added over time with the changes. This has led to the use of PRISMA but has less of a focus on engaging with stakeholders. Hence why the selected model was chosen and the lead author ensured a diverse selection of co-authors to feed into and nourish the selection of the final health models based on their collective experiences. Within the 2 ^nd^ study on this topic the authors will seek to include Prisma as a more focused population will increase the available literature. This point you raise was highlighted with the journal and our justification was approved at the point of publication.

 R6. We have sought to acknowledge/infuse your salient point within the beginning of the section on results. Again, thank you for your observation.    

**I think that a discussion section relating to the results and findings collectively is required.**

**Overall, this is a well written paper in large parts, but I think lacks some of the methodological detail I would expect to see. Stages of Scoping Review and how it is applied and the absence of  search terms and paper inclusion / exclusion criteria are missing and hit results. It is also missing the process used for determining what was included and rejects.  A Prisma diagram would somewhat attend to this and a write up of the process would be beneficial, this could be added in table form.**

**I think the results section requires some reorganisation to flow properly for the reader with a reformat and new subheadings.**

 R7. Please see our previous last comment. We acknowledge that structuring of any article can be altered and this is often to the preference of a reader (reviewer). Collectively, the authors feels that the structure does align itself to be understood by the reader whilst also remaining within the word count confines of the publication. We will certainly look to revisit this point within our next article linked to this subject matter.  

**I would suggest that Self-Management and Self-efficacy within the Inclusion of Health Models for Community Care – becomes an opener for the results section. This would then lead onto the  four models you will be reporting on from the literature. Each of the 4 models should then be clearly sub-headed and follow on as they do, but I would suggest you share how many papers informed each sections and hence are reported on in each section.**

 R8. Thank you for your observation and review. Thank you for agreeing that our focus on self-management an self-efficacy is an appropriate opener. We have included how this was achieved within the section and links into our rationale for the particular model of scoping review which does not include the statistical requirements that was adopted within later variations of scoping reviews. Our justification was the need to draw upon the lived ‘clinical’ experience and competency of stakeholders and co-authors. The models are sub-headed and there is a leading statement on page7 & 8 that identifies (prepares) the reader for the identified 4 models. Again, thank you for your insight.

**Finally Future Considerations: Stigma and Habitus, I would suggest could become your discussion section and where you report upon what you found that aligns to the 2 objectives. I think objective 1 is met via the paper, but I am not sure you have from the findings reported on how this meets objective 2 fully.  Discussion needs strengthening to answer the paper objectives fully.**

**Conclusion Section - Some of the conclusion details could go into the discussion and I would suggest a briefer conclusion to highlight headline findings.**

**I hope my reflections are useful for consideration if the above are attended to satisfactory. I think this is worthy of publication if the above can be satisfactorily addressed as its content is of interest and adds something new to the literature.**

 R.9 Thank you for your excellent insight in how the discussion and conclusion sections can be enhanced. With this in mind the authors have included a discussion section and sought to add the depth you rightly identify. This includes a magnification on how the article has furthered knowledge on both of the articles identified objectives.

 We have also shortened the conclusion. Thank you for these points and for the time it has taken you to review our article. On behalf of the authors, it is appreciated and acknowledged.

# Reviewer report: Reviewer response for version 2

**DOI:** 10.5256/f1000research.170940.r318620

**Published:** 2024-09-03

**Authors:** Mohamad Musa

**Affiliations:** 1 Cape Breton University, Sydney, Nova Scotia, Canada

The requested changes and edits were addressed by the authors.

Are the rationale for, and objectives of, the Systematic Review clearly stated?

Yes

Is the statistical analysis and its interpretation appropriate?

Yes

If this is a Living Systematic Review, is the ‘living’ method appropriate and is the search schedule clearly defined and justified? (‘Living Systematic Review’ or a variation of this term should be included in the title.)

Yes

Are sufficient details of the methods and analysis provided to allow replication by others?

Partly

Are the conclusions drawn adequately supported by the results presented in the review?

Partly

Reviewer Expertise:

Mental health, mental illness, the Middle East region, migrant and refugee health.

I confirm that I have read this submission and believe that I have an appropriate level of expertise to confirm that it is of an acceptable scientific standard.

# Response

**Published:** 2024-09-27

**Authors:** Dr. Richard Mottershead

**Affiliations:** College of Health Sciences, University of Sharjah, Sharjah, Sharjah, United Arab Emirates

On behalf of my co-authors and myself, I would like to thank the reviewer for their time and comments which we have assimilated into our article. 

 We have inserted a section to expand on your wish to provide more insight into the  methodology and use justification of adopting the use of a scoping review. We would suggest that this supports Table 1 and links well into sub section 'applied search strategy'.

 Lastly, we have included a discussion on the models of care which will believe enhances and strengthens the already present comments on the challenges and nuances of care within the MENA region. 

 Again, we collectively thank you for your input and review.

# Reviewer report: Reviewer response for version 1

**DOI:** 10.5256/f1000research.168133.r313321

**Published:** 2024-08-14

**Authors:** Mohamad Musa

**Affiliations:** 1 Cape Breton University, Sydney, Nova Scotia, Canada

This article presents a scoping review examining health models that could support the implementation of social prescribing and address the social determinants of mental health (SDMH) in community mental health care settings in the United Arab Emirates (UAE). The authors argue there is a need to move away from hospital-based mental health care toward more community-based approaches in the UAE and wider Middle East region. They review four health models - the Health Belief Model, Motivational Interviewing, Health Promotion Model, and Crescent of Care Nursing Model - and discuss how these could provide frameworks for implementing social prescribing to address SDMH in community sett

 The scoping review methodology is described, but more details could be provided on the specific search strategy, inclusion/exclusion criteria, and how articles were selected and analyzed. The authors mention using the Arksey and O'Malley framework but do not fully detail how each step was implemented.

 The authors provide a reasonable argument for how the reviewed health models could support implementation of social prescribing and address SDMH in community settings. However, the conclusions could be strengthened by more clearly linking the specific components of each model to social prescribing approaches and SDMH. Additionally, more discussion of potential limitations of the models or challenges in implementation would provide a more balanced assessment.

 Good work overall and the paper may be approved after the multiple revisions above.

Are the rationale for, and objectives of, the Systematic Review clearly stated?

Yes

Is the statistical analysis and its interpretation appropriate?

Yes

If this is a Living Systematic Review, is the ‘living’ method appropriate and is the search schedule clearly defined and justified? (‘Living Systematic Review’ or a variation of this term should be included in the title.)

Yes

Are sufficient details of the methods and analysis provided to allow replication by others?

Partly

Are the conclusions drawn adequately supported by the results presented in the review?

Partly

Reviewer Expertise:

Mental health, mental illness, the Middle East region, migrant and refugee health.

I confirm that I have read this submission and believe that I have an appropriate level of expertise to confirm that it is of an acceptable scientific standard, however I have significant reservations, as outlined above.
